# The changing landscape of anti-lymphoma drug clinical trials in mainland China in the past 15 years (2005–2020): A systematic review

**DOI:** 10.1016/j.lanwpc.2021.100097

**Published:** 2021-02-05

**Authors:** Haizhu Chen, Yu Zhou, Xiaohong Han, Yuankai Shi

**Affiliations:** a Department of Medical Oncology, National Cancer Center/National Clinical Research Center for Cancer/Cancer Hospital, Chinese Academy of Medical Sciences & Peking Union Medical College, Beijing Key Laboratory of Clinical Study on Anticancer Molecular Targeted Drugs, No. 17 Panjiayuan Nanli, Chaoyang District, Beijing 100021, China; b Clinical Pharmacology Research Center, Peking Union Medical College Hospital, Chinese Academy of Medical Sciences & Peking Union Medical College. No.41 Damucang Hutong, Xicheng District, Beijing 100032, China

**Keywords:** Lymphoma, Clinical trials, Anti-cancer drug, China

## Abstract

**Background:**

To depict a comprehensive changing landscape of anti-lymphoma drug clinical trials in mainland China from 2005 to 2020.

**Methods:**

A systematic review was conducted on the China National Medical Products Administration Center for Drug Evaluation platform, the Chinese Clinical Trial Registry and ClinicalTrials.gov websites.

**Findings:**

A total of 797 anti-lymphoma drug clinical trials registered from Jan 1st, 2005 to Aug 1st, 2020 were identified. The number of trials increased gradually over time, and a notable increase was observed in 2016, with the number growing from 29 in 2015 to 72 in 2016. Trials in phase I (26•1%) and phase II (26•6%) represented the majority, followed by phase III (12•5%) and phase IV (7•4%). Regarding sponsorship, industry-sponsored trials (53•2%) accounted for a slightly larger proportion than investigator-initiated trials (IITs) (46•8%). A dramatic growth for IITs was seen during 2017–2020, with the number increasing from 36 in 2017 to 96 in 2020. Additionally, the proportion of trials involving targeted agents (50•2%) accounted for the largest, followed by trials involving immunotherapy agents (41•0%), and cytotoxic agents (8•0%). Besides, a sustainable growth was observed in the number of leading anti-lymphoma drug clinical trial units in mainland China over the past 15 years. The majority of leading principal units (60•8%) were from Beijing, Shanghai, Guangdong and Jiangsu.

**Interpretation:**

In the past 15 years, the research and development of drugs and clinical trials for lymphoma in mainland China has achieved much progression. Future efforts are needed for improving innovation and sustainability of pharmaceutical research and development.

**Funding:**

China National Major Project for New Drug Innovation (2017ZX09304015); Chinese Academy of Medical Sciences (CAMS) Innovation Fund for Medical Sciences (CIFMS) (2016-I2M-1-001).

## Research in context

### Evidence before this study

We searched PubMed for papers with the terms “Lymphoma”, “Clinical trials”, and “China” for articles published from Jan 1st, 2005 to Aug 1st, 2020. There were three previous studies focusing on the whole landscape of anti-cancer drug clinical trials in China and two studies analysing CAR-T cell therapy in China. The available studies mainly focused on the whole landscape of all kinds of anti-cancer drugs research and development (R&D) in China, without specific attention on lymphoma. To our knowledge, the study of analysing and reviewing the development of anti-lymphoma drug clinical trials in China is lacking.

### Added value of this study

The rapid increase of newly registered clinical trials showed the progression of R&D in clinical trials for anti-lymphoma drugs through these years. The number of clinical trials increased gradually over the past 15 years, with a notable increase observed after 2016. The number of investigator-initiated trial has increased during 2011–2020. For industry-sponsored trial (IST), immunotherapy agents and targeted drugs have compromised over 80% of ISTs. CD20 monoclonal antibody and BTK inhibitor are the most commonly tested targeted drugs, and anti-PD-1 monoclonal antibody and CAR-T cell therapy have emerged as a new force for clinical trials of lymphoma in mainland China.

### Implications of all the available evidence

This systemic review provides evidence for the changing landscapes of anti-lymphoma drug clinical trials in the past 15 years (2005–2020) in mainland China. This study also suggests unmet clinical needs and the relevant data for clinicians, investigators, pharmaceutical enterprises, policy makers and other stakeholders, which can shed some light on future strategies in R&D of anti-lymphoma drugs.

## 1 Introduction

Cancer has become a major public health problem worldwide and is the leading cause of death in China, causing much disease burden [1]. Lymphoma, consisting of Hodgkin's lymphoma (HL) and non-Hodgkin's lymphoma (NHL), is one of the most common types of cancer in China and worldwide. It was estimated that there were 6900 new cases of HL and 68,500 of NHL in China in 2016, accounting for 9•5% of HL and 14•9% of NHL in the world [2]. Research showed that the mortality of lymphoma and myeloma increased in China from 2004 to 2017, with an estimated 52,000 deaths associated with lymphoma and myeloma occurring in 2017 [3].

Lymphoma is a heterogeneous malignancy and is mainly treated with drug therapy. Encouraging the research and development (R&D) of anti-lymphoma drugs is important for increasing the availability of new drugs and improving the survival outcomes for lymphoma patients. The Chinese government has issued a series of regulatory policies for promoting the development of innovative drugs and clinical trials since 2015 [4], [5], [6], [7]. A new landscape of R&D for anti-cancer drugs has emerged. Although there were some previous studies focusing on whole landscape of anti-cancer drug clinical trials and the development of specific treatment in China [8], [9], [10], [11], [12], to our knowledge, the analysis and review of anti-lymphoma drug clinical trials is lacking.

Therefore, we conducted this systematic review to analyze the trend of drug clinical trials for lymphoma in mainland China from 2005 to 2020. We also aimed to provide insight for the changing landscape in biopharmaceutical research, potency of investigator-initiated trial (IIT) and industry-sponsored trial (IST), unmet clinical needs and the supportive data for clinicians, investigators, pharmaceutical enterprises, policy makers and other stakeholders.

## 2 Methods

### 2.1 Data sources

A systematic review of registered clinical trials for lymphoma in mainland China was conducted on the Center for Drug Evaluation (CDE) of China National Medical Products Administration (NMPA) website (http://www.cde.org.cn/), the Chinese Clinical Trial Registry (ChiCTR) (http://www.chictr.org.cn) and ClinicalTrials.gov (https://clinicaltrials.gov/) websites. CDE registration platform is established for new drug registration trials. Registration on CDE registration platform is mandatory for new drug registration trials in China according to NMPA. Unlike CDE registration platform, ChiCTR receives registration of all interventional clinical trials and observational clinical trials. Detailed information of NMPA, CDE and ChiCTR can be found in Supplementary Material. In October 2004, the global clinical trial registration system was established and led by World Health Organization (WHO). Therefore, we chose Jan 1st, 2005 as the start date in this study. Trials initiated before this date and retrospectively registered were excluded from this study. The cut-off date of this study was Aug 1st, 2020. The study was in accordance with the Preferred Reporting Items for Systematic Reviews and Meta-Analyses (PRISMA) statement guidelines [13].

### 2.2 Selection criteria

A total of 399,218 clinical trial records registered on the three platforms were identified. We restricted the cancer type to lymphoma. The search term for CDE and ChiCTR was "lymphoma", and the registration information of 457 clinical trials potentially relevant to lymphoma were downloaded manually. We also searched ClinicalTrials.gov using the keyword "lymphoma" in the "Condition or disease" field, and restricted the country to China. Using this search strategy, 627 trials were identified from ClinicalTrials.gov website. Finally, a total of 1084 records were identified from three platforms.

To be eligible, clinical trials had to meet several criteria. Firstly, the trial must be intended for lymphoma and initiated after Jan 1st, 2005. Secondly, only trials involving anti-cancer drugs could be included. Adjuvant drugs, which were mainly for cancer supportive care and relieving side effects caused by anti-cancer therapy, such as painkillers, haemopoietic growth factors, and drugs for prevention and relief of nausea and vomiting, were excluded from this study. It was noteworthy that trials involving stem cell transplantation (SCT) were included. Thirdly, only trials of which study sites included mainland China were deemed eligible.

**Fig. 1:**
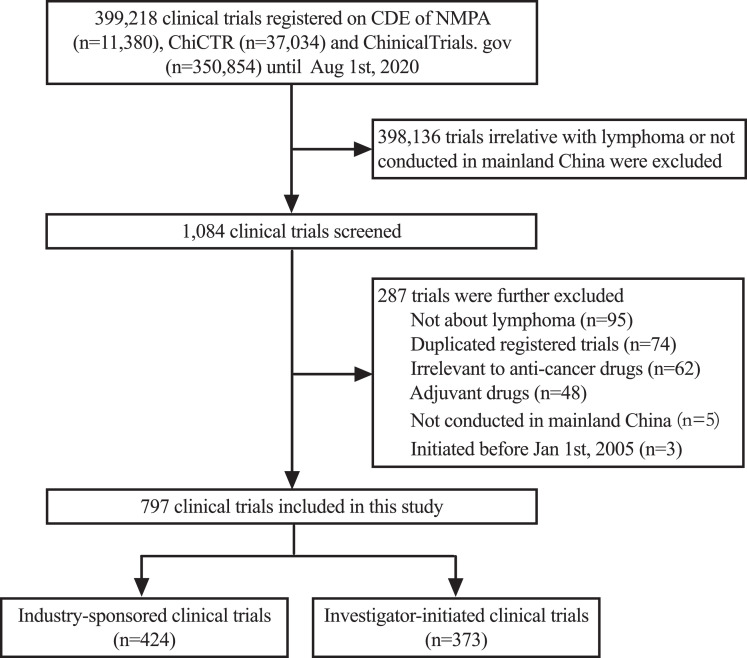
Flow diagram of the clinical trial selection process.

To identify the final data set for analysis, two investigators (HZC and YZ) independently reviewed the official title, cancer type, study design and study locations of the above 1084 trials. Disagreements about particular trials were discussed and resolved by consensus with all investigators. 287 trials were further excluded, including those not meeting the eligible criteria above, and those duplicately registered. Thus, a total of 797 anti-lymphoma drug clinical trials initiated between Jan 1st, 2005, and Aug 1st, 2020 were identified. The selection process is displayed in Fig. 1.

### 2.3 Data extraction and definition

Two investigators (HZC and YZ) independently extracted data from all the trials. Any discrepancy was resolved with the consensus of all investigators. The following characteristics were extracted for further analysis: trial identifier, sponsorship (ISTs or IITs), the phase of trial (I-IV, other), pathological subtype, primary tested drug, type and mechanism of drug, date of submission, number of participating centers, geographic region of leading unit, study design, recruitment status, key inclusion criteria, exclusion criteria, and primary and secondary endpoints.

ISTs are financially supported by the industry. Only drugs evaluated in ISTs were deemed as newly tested drugs, usually covering domestic innovative drugs, domestic biosimilars, generics, and global agents. IITs were defined as clinical trials proposed upon the initiative of clinical sponsor-investigators and without the company taking the role as a sponsor. Drug types were classified as cytotoxic agents, targeted agents, immunotherapy agents and SCT. Notably, targeted and immunotherapy drugs were sometimes not strictly mutually exclusive. Monoclonal antibodies, in a sense, belonged to both targeted agents and immunotherapy agents, whereas they were classified as targeted drugs in this study.

## 3 Statistical analysis

Descriptive statistics were performed to summarize the trial data with frequencies and percentages. Characteristics were compared using Chi-squared or Fisher's exact tests for categorical variables. We further analysed the time trends for specific indicators, comprising the number of registered initiated trials, the proportion of trials in each phase, the number of trials classified by sponsorship, the number of newly tested drugs, the number of trials involving different drug types and mechanism, and the number of leading clinical trial units. A two-sided *P* value of less than 0•05 was considered statistically significant. All statistical analyses were performed using the R software, version 3•6•2 (http://www.r-project.org/).

### 3.1 Role of the funding source

The funding source of this study do not influence or involve in study design; collection, analysis, and interpretation of data; the writing of the report; and the decision to submit the paper for publication. All authors had full access to the data and had final responsibility for the decision to submit for publication.

## 4 Results

### 4.1 Time trends of registered clinical trials

**Fig. 2:**
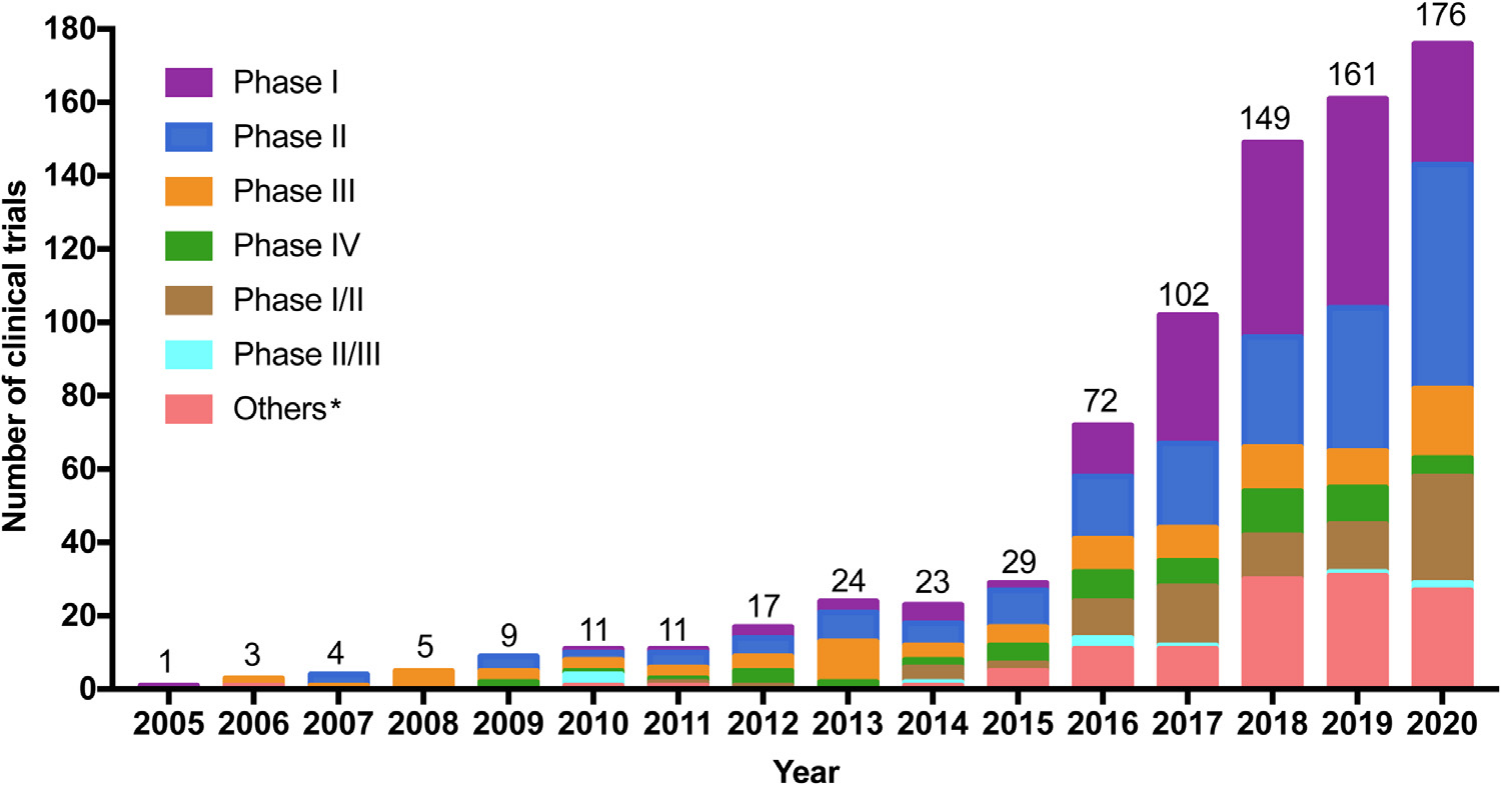
Annual numbers of anti-lymphoma drug clinical trials by study phase in mainland China during 2005–2020. Note: The data was from Jan 1st, 2015 to Aug 1st, 2020. * Others included 42 pilot studies, and 77 trials for which the phase information was unavailable on the registered platforms.

In total, 797 drug clinical trials conducted for lymphoma were registered on the three platforms from Jan 1st, 2005 to Aug 1st, 2020 in mainland China. 734 out of 797 (92•1%) clinical trials were domestic trials, of which 355 (48•4%) trials were conducted in single center and 379 (51•6%) in multiple centers. The remaining 63 (7•9%) trials were international multi-center trials. During 2005–2013, the number of clinical trials was extremely low, though it increased slowly over time (Fig. 2). Between 2013 and 2020, increasingly large numbers of clinical trials were registered, with a notable increase in 2016. The number of registered clinical trials had grown from 29 in 2015 to 72 in 2016, representing an increase of 148%. During the past decade (2011–2020), the number of trials increased substantially, with an average annual growth rate of 36•1%.

Regarding the distribution of study phase, phase Ⅰ and phase Ⅱ trials represented the majority proportion (52•7% [420/797]), with phase Ⅰ accounting for 26•1% (208/797) and phase Ⅱ accounting for 26•6% (212/797). For all 797 trials, a total of 100 (12•5%) trials was phase Ⅲ while 59 (7•4%) trials were phase IV, with 88 (11•0%) and 11 (1•4%) trials in phase Ⅰ/Ⅱ and phase Ⅱ/Ⅲ, respectively. Additionally, of 797 trials, 42 (5.3%) trials were pilot studies, and the phase information for 77 (9.7%) trials was unavailable on the registered platforms. Among the four different phases (phase Ⅰ-Ⅳ), the largest growth was observed in phase Ⅰ, followed by phase Ⅱ, phase Ⅳ and phase Ⅲ, with average annual growth rates of 47•5%, 35•4%, 22•8% and 19•6%, respectively.

### 4.2 Distribution of clinical trials by pathological subtypes

Diffuse large B cell lymphoma (DLBCL) is the most prevalent lymphoma subtype, comprising 33•27% of all lymphomas in Chinese population. When compared with Western countries, natural killer (NK)/T-cell lymphoma was more common, comprising 21•38% of all lymphomas in China, while HL and follicular lymphoma (FL) were less common [14]. Among the 797 registered trials in this study, no any specific pathological subtype was identified in 113 (14•2%) trials, while other 67 (8•4%) focused on NHL, 185 (23•2%) focused on B-cell NHL, and 23 (2•9%) focused on T-cell lymphoma, but without specific pathological subtypes reported. In the remaining trials, DLBCL was the most studied subtype (*n* = 178), followed by extranodal NK/T-cell lymphoma (*n* = 60), peripheral T-cell lymphoma (PTCL) (*n* = 51), chronic lymphocytic leukemia (CLL)/small lymphocytic lymphoma (SLL) (*n* = 33) and HL (*n* = 33). Only 14 trials involved marginal zone lymphoma, eight involved lymphoblastic lymphoma and two involved lymphoplasmacytic lymphoma. Detailed pathological subtypes identified are shown in Supplementary Fig. S1.

### 4.3 Distribution and time trends of clinical trials by sponsorship

**Fig. 3:**
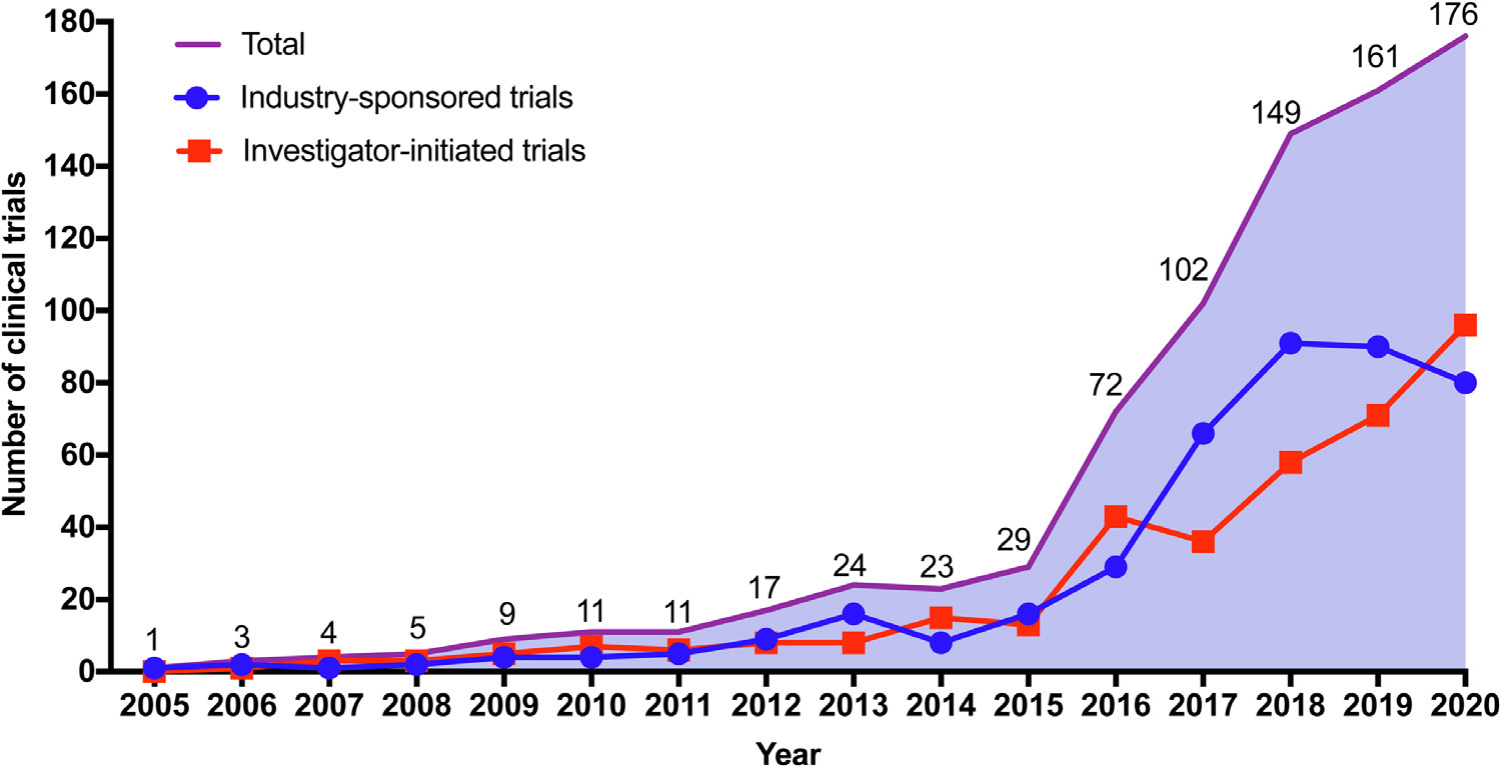
Annual numbers of anti-lymphoma drug clinical trials by sponsorship in mainland China during 2005–2020. Note: The data was from Jan 1st, 2005 to Aug 1st, 2020.

In terms of sponsorship, ISTs (53•2% [424/797]) accounted for a slightly larger proportion compared with IITs (46•8% [373/797]). All IITs were registered on ChiCTR or ClinicalTrials.gov platforms. Of 424 ISTs, 349 (82•3%) trials were sponsored by domestic pharmaceutical companies, and 75 (17•7%) trials were sponsored by overseas or multinational pharmaceutical enterprises. The number of ISTs increased gradually during 2011–2017, with an average annual growth rate of 53•7%, whereas it remained stable during 2018–2020. IITs are usually funded by academic institutions (i.e., hospitals or research institutes) and government. For clinical trials conducted on lymphomas in mainland China, the number of IITs displayed a continued increase during 2011–2020, with an average annual growth rate of 36•1%. A dramatic growth was seen during 2017–2020, with the number of IITs increasing from 36 in 2017 to 96 in 2020. Overall, IITs appeared quite active and played a vital role for anti-lymphoma drug clinical trials in mainland China. Details can be found in Fig. 3.

### 4.4 Time trends of newly tested drugs

Newly tested drugs investigated in ISTs were further analysed. From 424 ISTs, a total of 218 newly tested anti-lymphoma drugs were involved in clinical trials during 2005–2020. According to mechanism, 9•2% (20/218) of drugs were cytotoxic agents, with 46•3% (103/218) and 44•5% (97/218) of drugs belonging to targeted agents and immunotherapy drugs, respectively. The number of drugs tested in clinical trials increased over time, with an annually average growth rate of 40•2% during 2005–2020, and the accumulative total number had reached 218 at the cut-off date of Aug 1st, 2020. Compared with the accumulative total number of newly tested drugs by 2015 (*n* = 34), 184 new drugs were newly added between 2016 and 2020, representing an approximately 5•4-fold increase (Supplementary Fig. S2).

### 4.5 Distribution and time trends of clinical trials by drug type or mechanism

**Fig. 4:**
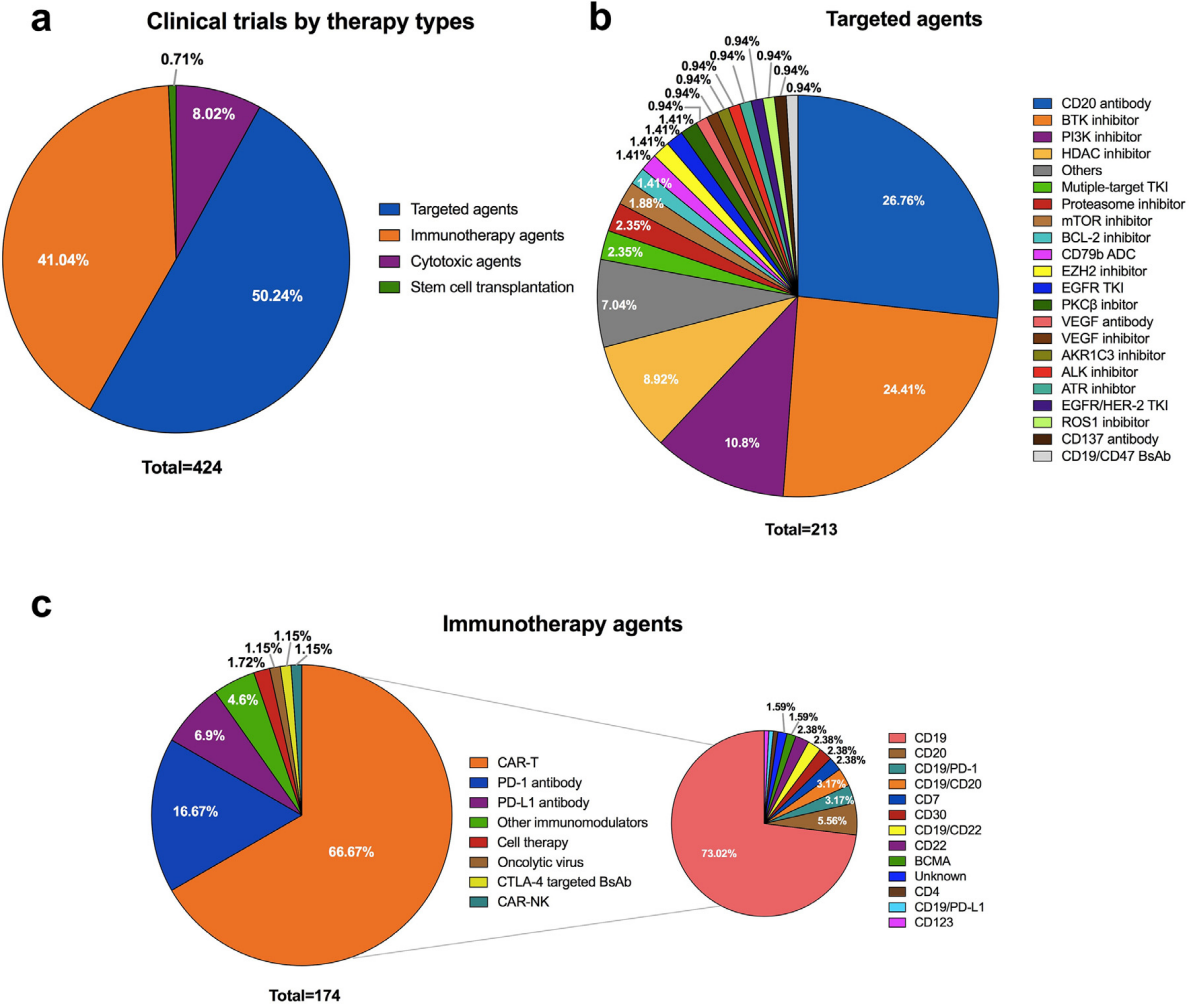
Distribution of clinical trials by drug types and mechanism in mainland China from Jan 1st, 2005 to Aug 1st, 2020. This analysis was done on industry-sponsored trials investigating newly tested drugs. a. Overview of clinical trials by drug types; b. Distribution of trials involving targeted agents; c. Distribution of trials involving immunotherapy. Abbreviations: BTK, bruton's tyrosine kinase; PI3K, phosphatidylinositol 3-kinase; HDAC, histone deacetylase; TKI, tyrosine kinase inhibitor; BCL-2, B-cell lymphoma-2; ADC, antibody-drug conjugate; EZH2, enhancer of zeste 2 polycomb repressive complex 2 subunit; mTOR, mammalian target of rapamycin; EGFR, epidermal growth factor receptor; PKCβ, protein kinase C-β; VEGF, vascular endothelial growth factor; AKR1C3, aldo-keto reductase family 1 member C3; ALK, anaplastic lymphoma kinase; ATR, ataxia telangiectasia and Rad3 related; HER-2, human epidermal growth factor receptor-2: ROS-1, c-ros oncogene 1 receptor tyrosine kinase; BsAb, bispecific monoclonal antibody; CAR-T, chimeric antigen receptor T cell; PD-1, programmed cell death-1; PD-L1, programmed cell death-ligand 1; CTLA-4, cytotoxic T lymphocyte associate protein-4; BCMA, B cell maturation antigen. Note: Among 116 registered trials involving CAR-T therapy, there are three trials investigating different kinds of CARs which targeted against one molecule, such as CD19, CD22, CD30, CD7, BCMA or CD123, etc.

**Fig. 5:**
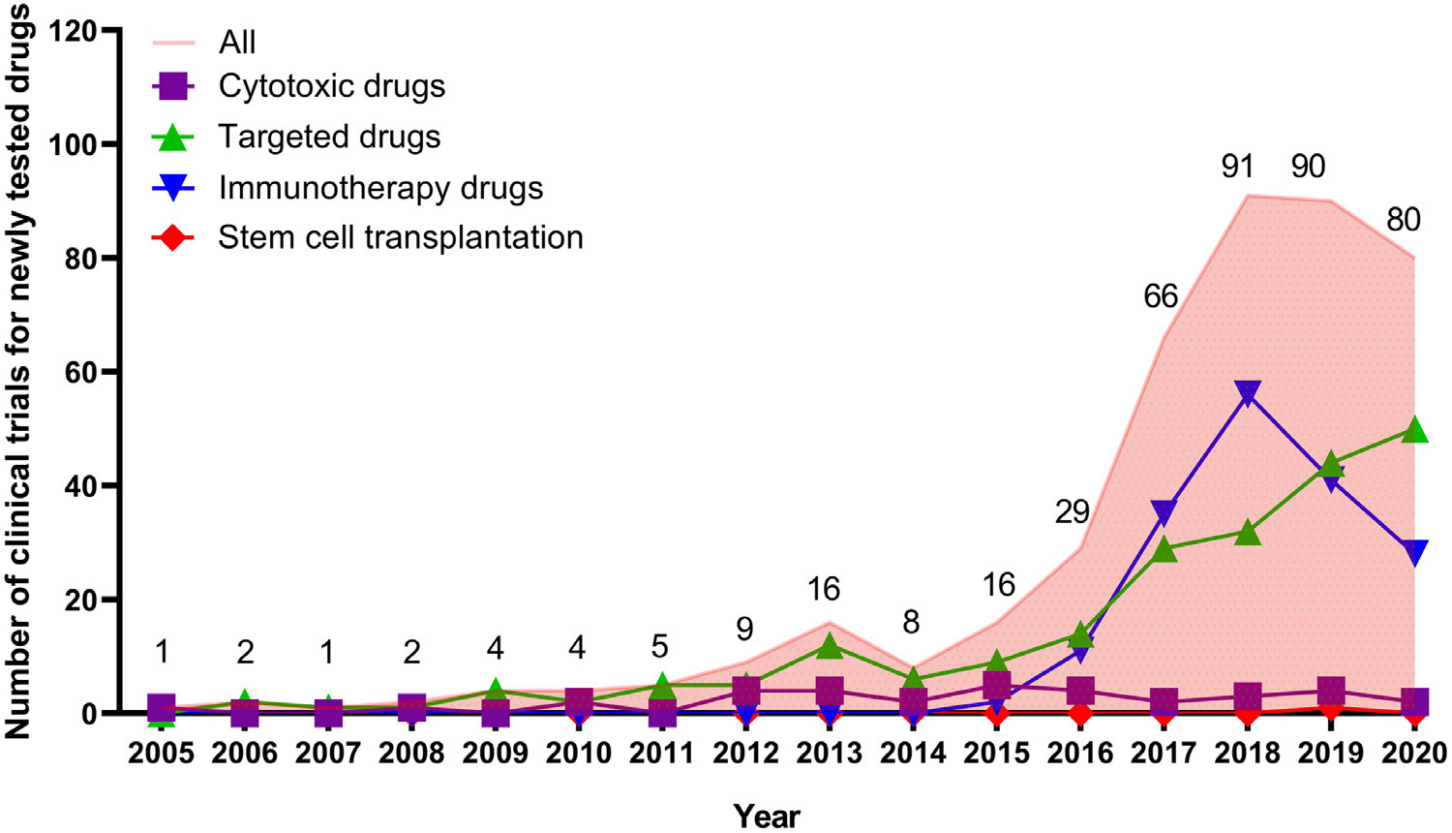
Annual numbers of clinical trials involving newly tested drugs in mainland China during 2005–2020. Note: This analysis was done based on industry-sponsored trials. The data was from Jan 1st, 2005 to Aug 1st, 2020.

Fig. 4 showed the distribution of clinical trials by drug types or mechanism, which was also analysed based on ISTs. In total, the proportion of trials involving targeted agents (50•2% [213/424]) accounted for the largest, followed by trials involving immunotherapy agents (41•0% [174/424]) and cytotoxic agents (8•0% [34/424]). Only three trials involved SCT. Since 2011, the number of trials involving targeted drugs had continued to increase with an average annual growth rate of 36•1%, and notable increase was observed during 2016–2020 (Fig. 5). The number of trials involving immunotherapy drugs remained scarce before 2015, and increased steeply during 2015–2018, with the highest number of 56 occurring in 2018. Of the 56 clinical trials, 34 (60•7%) were trials for chimeric antigen receptor T cell (CAR-T) therapy and 19 (33•9%) were trials for anti-programmed cell death-1 (PD-1)/ programmed cell death-ligand 1 (PD-L1) therapies. However, the number decreased from 56 in 2018 to 41 in 2019, with the number in 2019 slightly lower than that of trials involving targeted drugs in the same year (44 and 41 trials for targeted drugs and immunotherapy, respectively).

In terms of targeted therapy in lymphoma, CD20 remained the most studied target to date. Among the 213 trials investigating targeted agents, 57 (26•76%) investigated monoclonal antibodies against the CD20 B-cell antigen. Fifty-two (24•41%) clinical trials involved Bruton's tyrosine kinase inhibitors (BTKi), 23 (10•8%) involved phosphatidylinositol 3-kinase inhibitors (PI3Ki) and 19 (8•92%) involved histone deacetylase inhibitors (HDACi). In addition, bispecific antibody (BsAb), a promising treatment strategy, is an emerging field. There were three registered trials focusing on BsAb during 2019–2020, including two on CD3/CD19 (CTR20190205 and CTR20191955) and one on CD20/CD47 (CTR20192612).

As for immunotherapy, CAR-T cell therapy, a major source of immunotherapy for lymphoma in mainland China, represented the hottest strategy, with 116 of 174 (66•67%) trials evaluating it. CD19 was the most common CAR-T target, and there were 92 trials having CD19 as a target. Other CAR-T targets tested in clinical trials included CD20 (*n* = 7), CD7 (*n* = 3), CD30 (*n* = 3), CD22 (*n* = 3) and B cell maturation antigen (BCMA) (*n* = 2). Additionally, a total of 11 trials tested two CAR-T targets (i.e., CD19/CD22, CD19/CD20) either simultaneously or sequentially. PD-1/PD-L1 were also well-studied targets of immunotherapy in lymphoma, especially in HL. In 174 trials investigating immunotherapy, there were 29 (16•67%) and 12 (6•9%) trials targeting PD-1 and PD-L1, respectively, all of which were monoclonal antibodies. Also, BsAbs have emerged as a promising therapeutic strategy in cancer immunotherapy in recent years. There were two trials involving BsAbs for lymphoma in China to date, with one focusing on anti-cytotoxic T lymphocyte associate protein-4 (CTLA-4) /PD-1 (NCT04444141) and one on anti-CTLA-4/PD-L1 (NCT03733951).

### 4.6 Geographical distribution of clinical trials

**Fig. 6:**
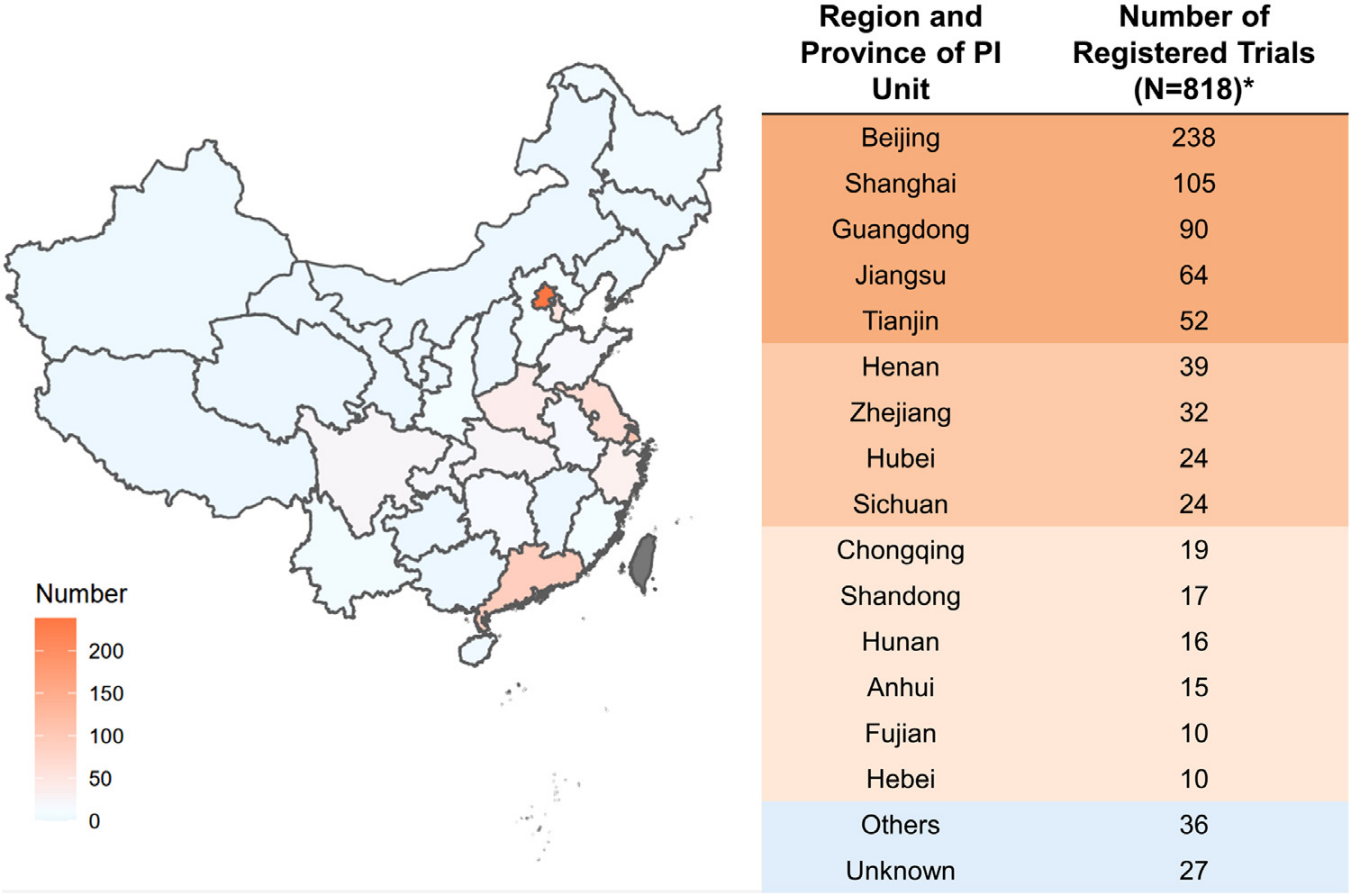
Distribution of trials according to region and province of principal investigator (PI) unit in mainland China during 2005–2020. *There are 21 registered trials with more than one PI unit.

Overall, 797 clinical trials were conducted at 25 different provinces or municipalities across China. 497 of 797 (60•8%) clinical trials were initiated by principal investigators (PI) from Beijing (*n* = 238), Shanghai (*n* = 105), Guangdong (*n* = 90) and Jiangsu (*n* = 64). By contrast, the number of trials led by PI from several provinces, such as Jilin (*n* = 3), Xinjiang (*n* = 2), Guizhou (*n* = 1) and Jiangxi (*n* = 1), were less than five, reflecting a severe uneven geographical distribution in clinical trials for lymphoma across China. Detailed geographical distribution of clinical trials is demonstrated in Fig. 6.

### 4.7 Time trends of leading clinical trial units

We further analysed time trends of leading clinical trial units during 2005–2020. Consistent with the increase in the number of clinical trials, the number of leading clinical trial units for lymphoma in mainland China also increased gradually, with the accumulative total number having reached 159 as of Aug 1st, 2020. Overall, the number of newly added leading clinical trial units was very limited before 2016, with the accumulative total number of only 38 by 2015. A substantial growth was identified during 2016–2020, with the number of newly added leading units over these four years reaching 121, corresponding to 76•1% of all leading units. The time trends of leading clinical trial units are illustrated in Supplementary Fig. S3.

### 4.8 Trend changes of characteristics of clinical trials

In 2015, the State Council of the People's Republic of China issued the policy "Opinions on reforming the examination and approval system for drug and medical devices" [4]. This policy is important for the development of clinical trials in China, which spurred drug innovation and optimized the drug clinical trial registration and application process. Thus, we further analysed the trend changes of characteristics of clinical trials by two temporal subsets: the first period 2005–2015, and the second period 2016–2020. Table 1 presents trend changes of characteristics of clinical trials between the two time periods. Regarding the study phase, 11•7% of trials were phase 1 during 2005-2015, compared to 29•1% during 2016-2020 (*P*<0.001). In addition, the proportion of domestic trials increased from 79•6% during 2005-2015 to 94•1% during 2016-2020 (*P*<0.001). Regarding the drug type, 5•9% of trials involved immunotherapy drugs during 2005-2015, compared to 48•2% during 2016-2020 (*P*<0.001). However, 27•9% and 4•2% of trials investigated cytotoxic agents during 2005-2015 and during 2016-2020 (*P*<0.001), respectively. This finding indicated that researchers shifted their focus from chemotherapy to immunotherapy.

**Table 1.** Trend change of characteristics of anti-lymphoma drug clinical trials in mainland China.

Characteristic	Total	Number of trials (%)		*P* value
		2005–2015	2016–2020	
	N (%)	N (%)	N (%)	
**Sponsorship**				
ISTs	424 (53•2)	68 (49•6)	356 (53•9)	0•358
IITs	373 (46•8)	69 (50•4)	304 (46•1)	
**Study phase**				
Phase I	208 (26•1)	16 (11•7)	192 (29•1)	<0•001
Phase II	212 (26•6)	42 (30•7)	170 (25•8)	
Phase III	100 (12•5)	41 (29•9)	59 (8•9)	
Phase IV	59 (7•4)	17 (12•4)	42 (6•4)	
Other	218 (27•4)	21 (15•3)	197 (29•8)	
**Number of centers**				
Single	357 (44•8)	57 (41•6)	300 (45•5)	0•410
Multicenter	440 (55•2)	80 (58•4)	360 (54•5)	
**Country**				
Domestic	734 (92•1)	109 (79•6)	625 (94•1)	<0•001
International	63 (7•9)	28 (20•4)	35 (5•3)	
Newly tested drug type*				
Cytotoxic agents	34 (8.1)	19 (27.9)	15 (4.2)	<0•001
Targeted agents	213 (50.6)	45 (66.2)	168 (47.6)	
Immunotherapy agents	174 (41.3)	4 (5.9)	170 (48.2)	

Abbreviations: ISTs, industry-sponsored trials; IITs, investigator-initiated trials.

⁎ Analysis was performed based on ISTs.

## 5 Discussion

Lymphoma is a heterogeneous disease that is usually approached with different treatment modalities, among which medication therapy is the most important therapeutic strategy for most lymphoma subtypes. The first-developed anti-cancer agent, nitrogen mustard was applied in the treatment for HL [15]. This resembles the start of medical oncology era. Till 1990s, the main treatment approach of medical oncology remained cytotoxic drug and lymphoma was one of the cancer types with good treatment result. In 1997, US Food and Drug Administration (FDA) approved the first unconjugated (naked) monoclonal antibody, rituximab, for the treatment of relapsed CD20 positive B-cell lymphoma, which marked a new era of targeted therapy [16]. In 2016, FDA approved nivolumab for the treatment of relapsed or refractory (r/r) classical HL (cHL), further changing the therapeutic scenario for lymphoma and setting up the new era of cancer immunotherapy [17]. From the scope of development in landmark drugs, the field of targeted therapy and immunotherapy in lymphoma treatments has been accelerating over the past decades and has been leading the forefront of precision medical oncology. Also, the scale of clinical trials in lymphoma was rapidly developing. From 2009 to 2018, lymphoma is one of the most studied cancer of clinical trials in China, for which the number of clinical trials ranked the second in all cancer types [11]. In some degree, the R&D of anti-lymphoma drugs and clinical trials represents the research capability of innovative hematology and oncology drugs. This systemic review provides information on the changing landscapes of anti-cancer drug clinical trials for lymphoma in the past 15 years (2005–2020) in mainland China. To the best of our knowledge, this is the first systematic review focusing on the landscape of anti-cancer drug clinical trials for lymphoma in mainland China through a long time period. The rapid increase of newly registered clinical trials showed the progression of biopharmaceutical R&D in lymphoma through these years.

The rapid growth of anti-lymphoma drug clinical trials is largely contributed to the policy support by Chinese government. Backlog of new drug application caused by long time of review and scarcity of domestic new drugs has hampered the development of anti-cancer drug innovation [18]. Since 2008, Chinese government has established national major project for new drug innovation, which stimulated the R&D of China's innovative new drug. To improve the procedure of review and approval for new drugs and medical devices, Chinese government has promulgated a series of reform policies since 2015 [4,[6], [7], [8]]. With these reform policies, the review procedure for innovation drugs has been prioritized. In 2017, NMPA became a regular member of International Council for Harmonization (ICH). The development of innovative drug in China was conducted according the standard of ICH. As a result, the numbers of newly tested anti-cancer drugs and clinical trials have increased rapidly since 2015, including lymphoma. There was a dramatic increase of phase I clinical trials for lymphoma between 2005 –2015 and 2016–2020 (Table 1). Also, the domestic anti-cancer drug clinical trials for lymphoma has increased since 2015. The increasing of clinical trials for lymphoma represents the effort of the Chinese government to improve the ability of pharmaceutical R&D, promote medical innovation and benefit lymphoma patients.

Immunotherapy agents and targeted drugs have compromised over 80% of industry sponsored clinical trials. Increasing trend of newly tested targeted drugs can be seen from 2015 to 2020. Among the targeted drugs, CD20 monoclonal antibody is the most commonly tested. The bloom of CD20 monoclonal antibody mainly attributed to the increasing investigation in the biosimilar of rituximab. In February 2015, CDE of NMPA launched “Guidelines for the research, development and technique assessment of biosimilar drugs (Trial)”, which accelerated the development of biosimilar drugs in China [6]. Since HLX01, the first biosimilar of rituximab, has been approved by NMPA in 2019 [19], the R&D of CD20 monoclonal antibody has become the hotspot for lymphoma. In March 2020, CDE asked public comments on “Guidelines of clinical trials in rituximab biosimilar drugs (the draft for comments)” [20]. This makes up the hiatus of the regulatory policies of clinical trials for rituximab biosimilar drugs and reflects the regulatory consideration for clinical trials in lymphoma.

Another hotspot for targeted drugs is BTKi, which accounted for 24•29% of targeted drugs in clinical trials for lymphoma. Zanubrutinib is a Chinese original innovative BTKi and is the first new drug simultaneously developed in both China and US [21]. Zanubrutinib was approved by FDA on Nov 15th, 2019 and approved by NMPA on Jun 3rd, 2020 for the treatment of mantle cell lymphoma [22]. The approval in both US and China was based on the data of a pivotal clinical trial conducted in China. The simultaneous development of innovative drugs can accelerate the approval procedure and bring more choices for accessibility of new drugs. With the improvement in regulatory and review system, joint review, and simultaneous approval of new drugs in multiple countries including China might become possible in the near future.

Another prosperous area of clinical trials for anti-lymphoma drug is immunotherapy. Numerous immunotherapeutic agents targeting PD-1, PD-L1, CTLA-4 have revolutionized treatment for cancer, including lymphoma. Among these, anti-PD-1 monoclonal antibody and CAR-T cell therapy are mostly investigated. On the one hand, anti-PD-1 monoclonal antibody has shown efficacy for the treatment of r/r cHL in recent clinical studies and has changed the treatment paradigm [23], [24], [25]. Based on the ORIENT-1 study [25], sintilimab became the first approved innovative anti-PD-1 monocloncal antibody for the treatment of r/r cHL in China. Besides monotherapy, anti-PD-1 monoclonal antibody is also under investigation for combination therapy. There are a variety of clinical trials exploring anti-PD-1 monoclonal antibody in other types of lymphoma, which can provide more treatment options for lymphoma patients in the future. On the other hand, CAR-T cell therapy has emerged as a new force in lymphoma treatment. Since the first CAR-T product tisagenlecleucel showed efficacy in leukemia and was approved for clinical treatment of refractory B cell acute lymphoblastic lymphoma by FDA, there were increasing numbers of clinical trials of CAR-T cells for the treatment of leukemia and lymphoma. To standardize and guide research, development, and registration for cell products, CDE of NMPA released “Guidelines principles for research and review of cell therapy products (Trial)” in Dec 22nd, 2017 [26]. This policy defined the declaration criteria of cell therapy as new drug. In Feb 24th, 2020, the new drug application (NDA) of FKC876 for the treatment of adult r/r DLBCL was accepted by CDE of NMPA, as the first CAR-T cell therapy under NDA review in China.

Besides the hotspots mentioned above, biopharmaceutical development has broadened the pipeline into newer attempts of clinical trials in lymphoma. First, more different types of lymphoma are under clinical trials, including some rare types with poor prognosis. For instance, results of this study showed that clinical trials for PTCL, an uncommon type of NHL which accounts for around 25% of NHL patients in China and about 10% in US and Europe [27,28], accounted for the third of all types of lymphoma. For r/r PTCLs, drugs including antifolate (pralatrexate), and HDACi (romidepsin, belinostat, vorinostat) are not accessible in mainland China and patients have few therapeutic options after relapse. In 2014, with promising data of a pivotal phase II study [29], the Chinese original innovative HDACi chidamide has been approved for the treatment of r/r PTCL. Recently, the result of a phase II clinical trial showed that an innovative anti-PD-1 antibody geptanolimab (GB226) has showed potent activity in r/r PTCL, which may provide new treatment approach for r/r PTCL patients [30]. Second, newer targets have emerged in registered clinical trials. For CAR-T cell therapy, CD20, CD7 and CD30 are newer CARs under investigation. For monoclonal antibodies, clinical trials targeting CD38, CD47, CD79b and CD137 are ongoing. Also, dual-target and multi-target inhibitors, BsAb and multi-targeted CAR-T cell therapies become the trends in lymphoma for clinical trials registered. Finally, simultaneous and sequential CAR-T cell therapy have entered the clinical trials. All these new developments indicate that the anti-cancer drug clinical trials of lymphoma in mainland China are making innovations, not simply replicating the success of those drug approved.

Since the first anti-cancer drug clinical trial of N-formyl botulinum toxin conducted in 1960s in China [31], the platform of anti-cancer drug clinical trials has developed during the last few decades. Up to Aug 1st, 2020, there are 1974 institutions as clinical trial unit approved by NMPA, including 888 institutions as clinical trial unit for anti-cancer drugs [32]. With the effort of Chinese government, academic institutions, and pharmaceutical enterprises, it is foreseeable that anti-lymphoma drug clinical trials in China will become an important part of treatment and provide more options for lymphoma patients in the future.

There are some concerns that should be addressed in the anti-cancer drug clinical trials for lymphoma. The geographic distribution of leading clinical trial unit across mainland China is uneven (Fig. 6). Additionally, the pipeline in China showed an uneven distribution among different immunotherapeutic drug classes, which is dominated by CAR-T cell therapy (66.67%). Also, novel study designs are warranted for clinical trials in lymphoma. Biomarker-based patient selection and precision medication can provide more potential benefit for lymphoma patients who participate in the clinical trials.

It is important to note that this study has limitation. The major limitation lies in the fact that monoclonal antibodies were directly classified into the targeted therapy category. We recognize that for some readers, monoclonal antibodies will be categorized as immunotherapy, given its mechanism of action such as antibody-dependent cell-mediated cytotoxicity or antibody-dependent phagocytosis, etc. However, we wanted to emphasize the role of CAR-T and other cell therapies as a main responsible source for anti-lymphoma drug clinical research growth in mainland China. Another limitation is that the ChiCTR registration platform was established in 2007 and CDE of NMPA registration platform was established in 2013. Although the registration of clinical trials was mandatory since 2007 according to WHO International Clinical Trials Registry Platform requirement and the Declaration of Helsinki (2008), the clinical trials that finished before 2007 and not registered retrospectively might have been omitted. Despite that the limited number of clinical trials from 2005 to 2007 would not have impact on the overall result and conclusion of this study, caution must be applied when interpretation of this result.

In conclusion, this systematic review provides an overview of anti-lymphoma drug clinical trials in mainland China from 2005 to 2020. With the rapid capability of clinical development and the sustained policy support from Chinese government, dramatic progress has been achieved in the R&D of anti-lymphoma drugs in the past 15 years in mainland China. Increasingly large numbers of clinical trials were conducted for lymphoma, and several representative drugs have been successfully approved in mainland China, which will further benefit Chinese lymphoma patients and make contributions for the global drug pipeline. Future efforts are needed for improving innovation and sustainability of pharmaceutical R&D.

## Data sharing statement

All data analysed during this study are included in this article. No additional data are available.

*Editor note: The Lancet Group takes a neutral position with respect to territorial claims in published maps and institutional affiliations.*

## Author Contributions

YKS and XHH contributed to the study concept and design. YKS, XHH, HZC and YZ contributed to data quality control and data interpretation. HZC and YZ performed the systematic search, data extraction and data analysis. YKS, XHH, HZC and YZ drafted and revised the manuscript. All authors confirm that they have full access to all the data in the study and accept responsibility to submit for publication.

## Declaration of Interests

All authors declare no competing interests.

## Appendix Supplementary materials

Image, application 1

Image, application 2
