# Supplementary material for: The changing landscape of anti-lymphoma drug clinical trials in mainland China in the past 15 years (2005–2020): A systematic review
Source: Lancet Reg Health West Pac. 2021 Feb 5;8:100097. doi: 10.1016/j.lanwpc.2021.100097 (PMC8315394; doi:10.1016/j.lanwpc.2021.100097)

**SUPPLEMENTAL MATERIAL**

**The changing landscape of anti-lymphoma drug clinical trials in mainland China in the past 15 years (2005-2020): a systematic review**

Haizhu Chen, Yu Zhou, Xiaohong Han and Yuankai Shi

[Supplemental information](#_Toc7651) 2

[Fig. S1 Distribution of drug clinical trials on lymphoma in mainland China during 2005-2020.](#_Toc7008) 3

[Fig. S2 Annual newly added and accumulative total numbers of newly tested anti-lymphoma drugs in mainland China during 2005-2020.](#_Toc27931) 4

[Fig. S3 Annual newly added and accumulative total numbers of leading anti-lymphoma drug clinical trial units in mainland China during 2005-2020..](#_Toc27931) 5

**Supplementary information**

*Introduction of databases*

National Medical Products Administration (NMPA) Center for Drug Evaluation (CDE)

NMPA, formerly named China Food and Drug Administration (CFDA), is the China’s agency for regulating drugs and medical devices. CDE, one of the main sectors of the NMPA, plays a critical role in the drug-approval process and the website of CDE offers feasible and reliable information for clinical trials. The website of CDE was established in Nov 1^st^, 2012 and put into practice in Nov 19^th^, 2012. In Sep 6^th^, 2013, cNMPA stated that all new drug registration trials in China must be registered on CDE registration platform, and trials initiated before 2013 but without the completion of new drug application had to be registered retrospectively.

The database can be accessed through <http://www.cde.org.cn/>.

Chinese Clinical Trial Registry (ChiCTR)

ChiCTR, a non-profit organization, was established in 2005 by West China Hospital, Sichuan University according to both the International Clinical Trials Register Platform (ICTRP) Standard of World Health Organization (WHO) and Ottawa Group Standard. The Ministry of Health of People’s Republic of China assigned ChiCTR to be the representative of China to join WHO ICTRP in 2007. ChiCTR has the largest number of registered clinical trials in China, and provides the services including registration for clinical trials. Unlike CDE registration platform, ChiCTR receives registration of all interventional studies and observational studies. Therefore, information of the investigator-initiated study along with industry sponsored study can be found in ChiCTR platform. The registration of first clinical trial on this platform was done on Sep 26^th^, 2005, and the first anti-cancer drug trials was registered on June 28^th^, 2007. According to the Declaration of Helsinki (2008), any clinical study involving human should be registered at public clinical registration platform. Thus, ChiCTR also accepted retrospective registration as of 2007.

The database can be accessed through <http://www.chictr.org.cn>.

**Fig. S1.** **Distribution of drug clinical trials on lymphoma in mainland China during 2005-2020.** SLL/SLL, chronic lymphocytic leukemia/small lymphocytic lymphoma; EBV, Epstein-Barr virus.

Note: There are 24 clinical trials involving more than one pathological type.


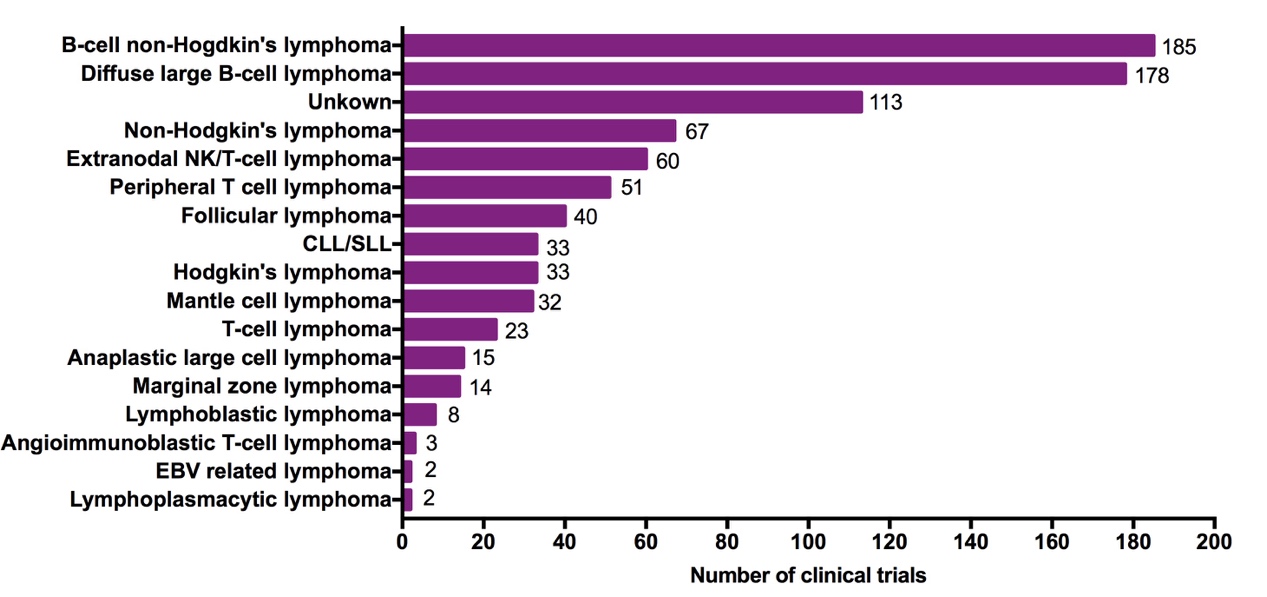


**Fig. S2. Annual newly added and accumulative total numbers of newly tested anti-lymphoma drugs in mainland China during 2005-2020.**

Note: The data cut-off date was Aug 1st, 2020.


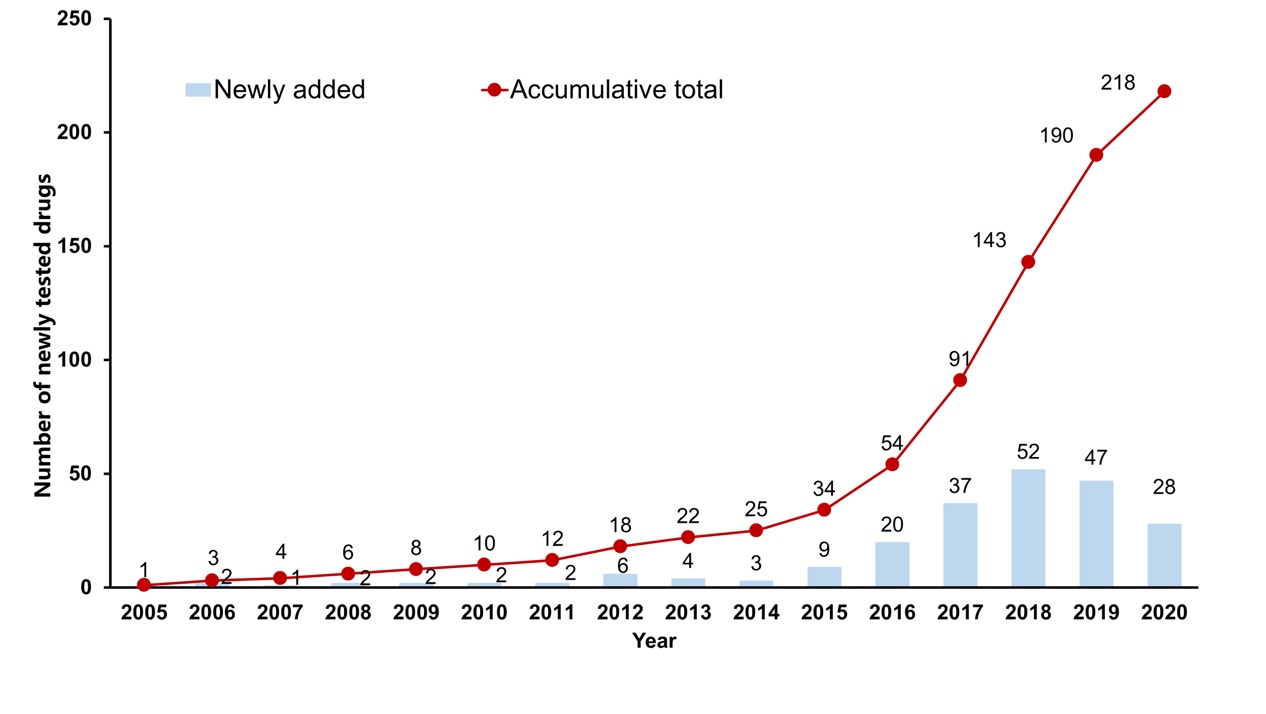


**Fig. S3. Annual newly added and accumulative total numbers of leading anti-lymphoma drug clinical trial units in mainland China during 2005-2020.**

Note: The data cut-off date was Aug 1st, 2020.


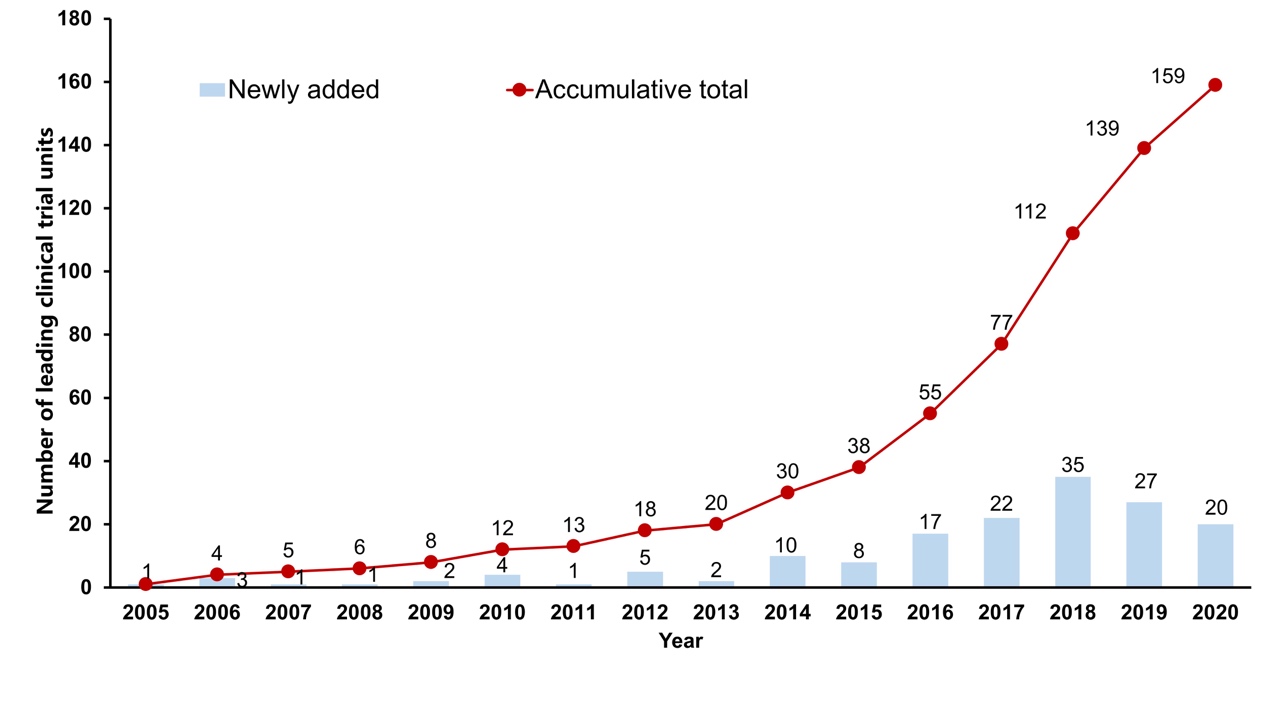

Supplement: Supplementary file 1 [file mmc1.docx]
