# Supplementary material for: The changing landscape of anti-lymphoma drug clinical trials in mainland China in the past 15 years (2005–2020): A systematic review
Source: Lancet Reg Health West Pac. 2021 Feb 5;8:100097. doi: 10.1016/j.lanwpc.2021.100097 (PMC8315394; doi:10.1016/j.lanwpc.2021.100097)
Supplement: Supplementary file 2 [file mmc2.docx]

**Abstract in Chinese**

**中国抗淋巴瘤药物临床试验15年（200****5-2020）：一项系统综述**

**陈海珠^1#^，周钰^1#^，韩晓红^2*^，石远凯^1*^**

**1. 国家癌症中心/国家肿瘤临床医学研究中心/中国医学科学院北京协和医学院肿瘤医院内科；抗肿瘤分子靶向药物临床研究北京市重点实验室，北京，100021**

**2. 中国医学科学院北京协和医院临床药理研究中心，北京，100032**

**目的**：分析2005年至2020年中国抗淋巴瘤药物临床试验的发展趋势。

**方法：**利用中国国家药品监督管理局药品审评中心平台、中国临床试验注册中心网站和ClinicalTrials.gov网站开展了一项系统综述。

**结果：**本研究共纳入2005年1月1日至2020年8月1日总计797项抗淋巴瘤药物注册临床试验。结果显示，抗淋巴瘤药物注册临床试验的数量随时间推移逐渐增加，在2016年出现显著增加，从2015年的29项增加至2016年的72项。Ⅰ期临床试验和Ⅱ期临床试验占到了试验总体的绝大多数，分别为26.1%和26.6%，其次是Ⅲ期临床试验（12.5%）和Ⅳ期临床试验（7.4%）。在发起者方面，企业发起的临床试验比研究者发起的临床试验占比稍多（53.2%比46.8%）。2017年至2020年，研究者发起的临床试验显著增加，从2017年的26项增加至2020年的96项。在药物类型方面，研究最多的依次为靶向治疗药物（50.2%）、免疫治疗药物（41.0%）和细胞毒药物（8.0%）。另外，在过去15年间，抗淋巴瘤药物临床试验主要研究者所在单位的数量持续增多，主要研究者所在单位依次分布在北京、上海、广东和江苏，占全部研究单位的60.8%。

**结论：**在过去15年间，中国抗淋巴瘤药物临床试验取得了长足进展，未来仍然需要更多的努力，以提高抗淋巴瘤药物研发的创新性、促进抗淋巴瘤药物临床试验的可持续发展。

**基金：**重大新药创制科技重大专项（2017ZX09304015）；中国医学科学院医学与健康科技创新工程（2016-I2M-1-001）

*Disclaimer: This translation in Chinese was submitted by the authors and we reproduce it as supplied. It has not been peer reviewed. Our editorial processes have only been applied to the original abstract in English, which should serve as reference for this manuscript.*
